# Disparities in positive mental health of sexual and gender minority adults in Canada

**DOI:** 10.24095/hpcdp.44.5.01

**Published:** 2024-05

**Authors:** Sonia Hajo, Colin A. Capaldi, Li Liu

**Affiliations:** 1 Centre for Surveillance and Applied Research, Public Health Agency of Canada, Ottawa, Ontario, Canada; 2 Department of Epidemiology, Biostatistics and Occupational Health, McGill University, Montral, Quebec, Canada

**Keywords:** sexual orientation, gender identity, health inequalities, positive mental health, life satisfaction, happiness, psychological well-being, community belonging

## Abstract

**Introduction:**

The goal of this study was to examine potential disparities in positive mental health (PMH) among adults in Canada by sexual orientation and gender modality.

**Methods:**

Using 2019 Canadian Community Health Survey (CCHS) Annual Component data (N=57034), we compared mean life satisfaction and the prevalence of high self-rated mental health (SRMH), happiness and community belonging between heterosexual and sexual minority adults, and between cisgender and gender minority adults. We used 2019 CCHS Rapid Response on PMH data (N = 11486) to compare the prevalence of high psychological well-being between heterosexual and sexual minority adults. Linear and logistic regression analyses examined the between-group differences in mean life satisfaction and the other PMH outcomes, respectively.

**Results:**

Sexual minority (vs. heterosexual) adults reported lower mean life satisfaction (B=−0.7, 95% CI: −0.8, −0.5) and were less likely to report high SRMH (OR = 0.4, 95%CI:0.3,0.5), happiness (OR = 0.4, 95% CI: 0.3, 0.5), community belonging (OR= 0.6, 95% CI:0.5, 0.7) and psychological well-being (OR = 0.4, 95% CI: 0.3, 0.6). Differences were not always significant for specific sexual minority groups in sex-stratified analyses. Gender minority adults reported lower mean life satisfaction and were less likely to report high SRMH and happiness than cisgender adults.

**Conclusion:**

Future research could investigate how these PMH disparities arise, risk and protective factors in these populations, how other sociodemographic factors interact with sexual orientation and gender identity to influence PMH and changes in disparities over time.

**Highlights**

- We investigated disparities in positive mental health (PMH) between sexual minority and heterosexual adults and between gender minority and cisgender adults in Canada in 2019.
- Mean life satisfaction was significantly lower among sexual minority adults than among heterosexual adults.
- Prevalence of high self-rated mental health, happiness, community belonging and psychological wellbeing were also significantly lower among sexual minority adults than among heterosexual adults.
- Mean life satisfaction and prevalence of high self-rated mental health and happiness were also significantly lower among gender minority adults than among cisgender adults.

## Introduction

In 2015–2018, 3.2% of individuals in Canada aged 15 years and older identified as gay, lesbian or bisexual,^1^ while in 2021, 0.3% identified as transgender or nonbinary.^2^ Sexual orientation and gender modality (i.e. the congruence or incongruence between gender identity and sex assigned at birth^3^) are sociodemographic characteristics that can have wide-ranging implications for health.^4^,^5^ Research shows that Two-Spirit, lesbian, gay, bisexual, transgender, queer, and additional people who identify as part of sexual and gender diverse communities (2SLGBTQ+)* individuals are at greater risk of negative psychological outcomes compared to their heterosexual and cisgender peers,^6^,^7^ including higher prevalence rates of depression and anxiety disorders, and of suicidal ideation and attempts among sexual minority^6^,^8^-^10^ and transgender^7^ individuals. Non-suicidal self-injury is also more prevalent among sexual and gender minority (SGM) people.^11^ Disparities in negative psychological outcomes between sexual minority and heterosexual people have also been observed in Canadian population health surveys.^12^-^16^

These inequalities are often explained using minority stress theory—namely, that SGM people experience worse mental health on average due to the excess stress caused by the stigma, prejudice and discrimination they face and by the internalization of negative societal attitudes.^17^,^18^ Supporting this theory, stigmatizing events, internalized transphobia or homophobia, expectations of rejection and identity concealment have been associated with negative psychological outcomes among 2SLGBTQ+ individuals.^19^-^22^

While existing research may provide insights into the experience of mental illness and distress in 2SLGBTQ+ populations, these outcomes do not encompass all aspects of mental health. The World Health Organization defines health as “a state of complete physical, mental and social well-being and not merely the absence of disease or infirmity.”^23^ This definition implies that mental health spans beyond mental illness (i.e. *ill*-being) and emphasizes the importance of the positive aspects of mental health (i.e. *well*-being). Based on the dual-continuum model of mental health, positive mental health (PMH) and mental illness do not fall on opposing ends of a single continuum but are distinct (albeit related) constructs.^24^,^25^ Accordingly, an individual may live with a mental illness, but still have relatively high PMH.^26^ Thus, to fully understand the mental health of SGM people, it is important to also examine their emotional, psychological and social well-being.^27^ A focus on PMH can move us beyond traditional biomedical and deficit-based approaches to a more strengths-based understanding of the mental health of SGM individuals.^28^

Some previous studies have investigated the overall PMH of sexual minority people in Canada using data from large population health surveys.^13^,^29^,^30^ For instance, analyses of data from the 2012 Canadian Community Health Survey (CCHS) – Mental Health indicates that sexual minority individuals had lower PMH than heterosexual individuals,^29^,^30^ while analyses from the 2015 CCHS only showed significant disparities in PMH among bisexual individuals.^13^ These studies examined PMH as a single broad construct in analyses; however, multiple PMH outcomes can be investigated to obtain a more fine-grained and nuanced understanding of different aspects of well-being in and between populations.^31^-^33^ Indeed, the Public Health Agency of Canada (PHAC) monitors the PMH of adults in Canada using five outcomes in its Positive Mental Health Surveillance Indicator Framework (PMHSIF): self-rated mental health (SRMH), happiness, life satisfaction, psychological well-being and community belonging.^31^,^32^

More generally, little is known about the PMH of gender minority individuals in Canada given that questions distinguishing between sex at birth and gender identity only began to be included in more recent population health surveys.^34^

Finally, there are indications that some PMH outcomes like high SRMH have been decreasing in prevalence in Canada since 2015;^35^ inequalities in PMH outcomes may have changed if temporal trends were not identical in both 2SLGBTQ+ and non-2SLGBTQ+ populations. To address these gaps, we used more recent data from 2019 to comprehensively examine disparities in PMH by sexual orientation and by gender modality across PMH outcomes from the PMHSIF.^31^,^32^

*Canada’s first Federal 2SLGBTQI+ Action Plan (https://women-gender-equality.canada.ca/en/free-to-be-me/federal-2slgbtqi-plus-action-plan/federal-2slgbtqi-plus-action-plan-2022.html) was launched in 2022 to improve data collection, analysis, research and knowledge on 2SLGBTQI+ people in Canada. Consistent with Statistics Canada’s approach (https://www.statcan.gc.ca/o1/en/plus/4313-improving-data-2slgbtq-populations), the acronym “2SLGBTQ+” is being used here rather than “2SLGBTQI+”, as this was the scope of the survey at the time.

## Methods

***Data and participants***

Data for four out of five PMH outcomes came from the 2019 CCHS Annual Component,^34^ which was collected from January to December 2019. The fifth PMH outcome, psychological well-being, was measured in the Rapid Response component of the 2019 CCHS,^36^ administered to respondents who participated in January to March 2019. Statistics Canada excluded from the target population of the 2019 CCHS full-time members of the Canadian Armed Forces as well as individuals living on First Nations reserves and other Indigenous settlements in the provinces, in institutions, in foster care if aged 12 to 17 years, or in two specific health regions in Quebec; less than 3% of individuals in Canada aged 12 years and older are represented in these exclusions. Individuals living in the territories were excluded from the target population for the Rapid Response component; territorial data were collected but unavailable in the 2019 Annual Component as these data are only representative after 2 years of data collection. The sampling frame used for the Labour Force Survey was also used in the 2019 CCHS for adults living in the provinces.

**Figure 1:**
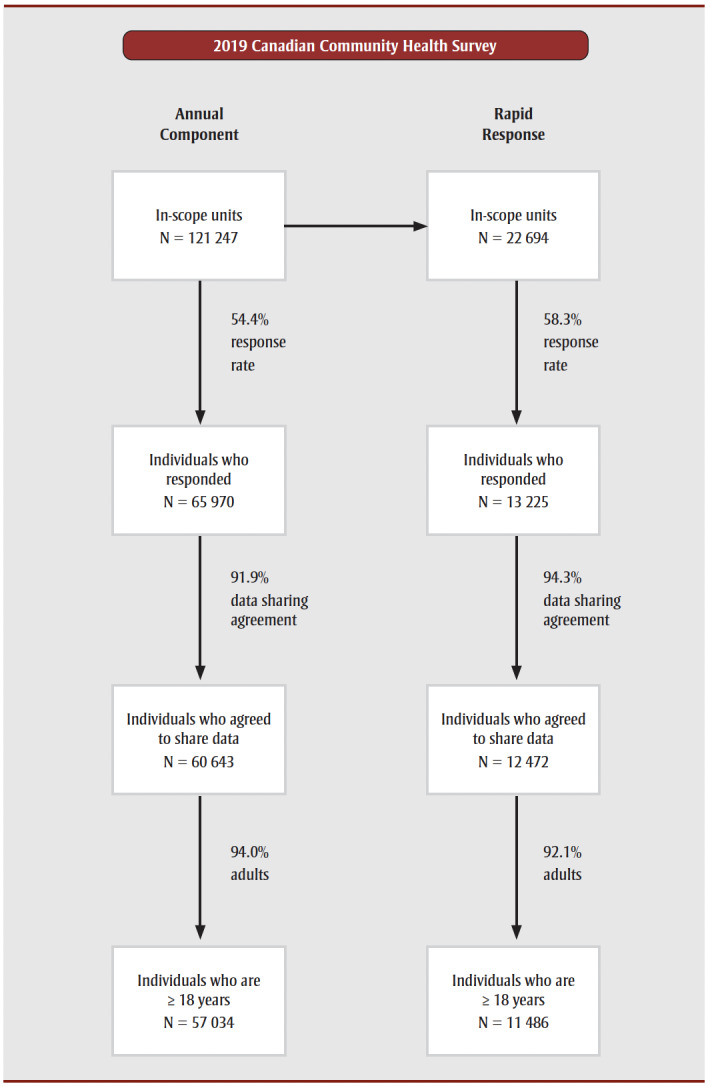
Flow chart depicting sample size reductions in the 2019 Canadian Community Health Survey Annual Component and Rapid Response component

Respondents completed the 2019 CCHS via computer-assisted telephone or in-person interviews. The 2019 CCHS collected data from individuals aged 12 years and older, although only those aged 15 years and older were asked about their sexual orientation. Nevertheless, we excluded youth aged 12 to 17 years from our study because different measures are used to monitor some indicators of PMH in the youth version of PMHSIF.^32^ Moreover, we only had access to data from respondents who agreed to share their data with PHAC and Health Canada. As illustrated in Figure 1, these restrictions led to sample sizes of 57034 for the Annual Component and 11486 for the Rapid Response.

***Measures***

**Positive mental health outcomes**

Of the five PMH outcomes included in the adult PMHSIF,^32^,^33^ SRMH, happiness, life satisfaction and community belonging were captured in the Annual Component. Psychological well-being was only measured in the Rapid Response. Our coding of high PMH was based on the cut-offs used in the PMHSIF.^32^

SRMH was assessed with the question, “In general, would you say your mental health is…?” Response options included “excellent,” “very good,” “good,” “fair” and “poor.” We dichotomously coded individuals who responded “excellent” or “very good” as having high SRMH. This type of question has been recommended as a measure of general mental health status by the OECD.25 Responses to this question have been associated with a wide range of physical and mental health outcomes.^37^

Happiness was assessed with the question, “Would you describe yourself as being usually...?” Response options included “happy and interested in life,” “somewhat happy,” “somewhat unhappy,” “unhappy with little interest in life” and “so unhappy, that life is not worthwhile.” We dichotomously coded individuals who responded “happy and interested in life” as having high levels of happiness.

Life satisfaction was assessed with the question, “Using a scale of 0 to 10, where 0 means ‘very dissatisfied’ and 10 means ‘very satisfied,’ how do you feel about your life as a whole right now?” In the current research we treated this as a numerical variable and report on mean life satisfaction. Happiness and life satisfaction are core aspects of hedonic well-being or the positive feeling component of PMH.^27^,^38^

Community belonging was assessed with the question, “How would you describe your sense of belonging to your local community? Would you say it is…?” Response options included “very strong,” “somewhat strong,” “somewhat weak” and “very weak.” We dichotomously coded individuals who responded “very strong” or “somewhat strong” as having high community belonging. This question captures the social integration aspect of social well-being, which can be considered part of eudaimonic well-being or the positive functioning component of PMH (along with psychological well-being).^27^,^38^

Psychological well-being was measured using the six items from the psychological well-being subscale of the Mental Health Continuum—Short Form.^39^ Respondents were asked how often in the past month they felt (1) that they liked most parts of their personality; (2) good at managing the responsibilities of their daily life; (3)that they had warm and trusting relationships with others; (4) that they had experiences that challenged them to grow and become a better person; (5) confident to think or express their own ideas and opinions; and (6) that their life had a sense of direction or meaning to it. These questions are designed to measure the six components of psychological well-being identified by Ryff^40^: self-acceptance, environmental mastery, positive relations with others, personal growth, autonomy and purpose in life. We recoded the following response options to represent the number of days in the past month: “every day“ (28days = 7 days per week 4 weeks); “almost every day” (20 days = 5 days per week 4 weeks); “about 2 or 3 times a week” (10 days = 2.5 days per week 4 weeks); “about once a week” (4 days = 1day per week 4 weeks); “once or twice” (1.5 days) and “never” (0 days).41 We averaged the recoded responses and dichotomously coded respondents with a mean score of 20 or higher as having high psychological well-being.

**Sexual orientation**

Respondents were asked, “What is your sexual orientation?” Response options were “heterosexual,” “homosexual,” “bisexual” and “please specify.” Individuals who specified a sexual orientation that could be classified as one of the existing response options were recoded into that category by Statistics Canada. We coded individuals who identified as homosexual (gay/lesbian), bisexual/pansexual or another sexual orientation as a sexual minority.

**Gender modality**

Respondents were asked, “What was your sex at birth?” Response options included “male” and “female.” This was followed by the question, “What is your gender?” Response options included “male,” “female” and “please specify.” When responses for sex at birth and gender were the same, we coded the individual as cisgender; when responses differed, we coded the individual as a gender minority.

**Analysis**

Using Annual Component data, we estimated mean life satisfaction and the percentage of high SRMH, happiness and community belonging by sexual orientation (heterosexual or sexual minority) and gender modality (cisgender or gender minority). We also obtained overall and sex-stratified estimates of these PMH outcomes for specific sexual minority groups (i.e. gay/lesbian and bisexual/pansexual); we did not separately report on PMH outcomes among those who identified as having another sexual orientation beyond heterosexual, gay/lesbian or bisexual/pansexual given the difficulty in interpreting findings for such a heterogeneous group.

We estimated the percentage of high psychological well-being using the Rapid Response data for individuals by sexual orientation (heterosexual or sexual minority). We also obtained overall and sex-stratified estimates of high psychological well-being for specific sexual minority groups (i.e. gay/lesbian and bisexual/pansexual). The estimate of psychological well-being for gender minority adults is not reported because it was not releasable (i.e. coefficient of variation [CV] > 35).

To determine whether the above estimates were significantly different, we conducted logistic regression analyses for the dichotomized PMH outcomes and linear regression analyses for life satisfaction. We used dummy coding for the linear regression analyses so that—similar to the logistic regression analyses—“heterosexual adults” was the reference group in the sexual orientation analyses and “cisgender adults” was the reference group in the gender identity analyses. We interpreted odds ratios (OR) with 95% confidence intervals (CIs) that did not include 1.0 as statistically significant in the logistic regression analyses. We interpreted coefficients with 95% CIs that did not include zero as statistically significant in the linear regression analyses.

For the overall comparisons of sexual minority adults to heterosexual adults, we also statistically controlled for a number of sociodemographic characteristics in follow-up logistic regression analyses for the dichotomized PMH outcomes and linear regression analyses for life satisfaction. Covariates included the individual’s sex at birth, age group, marital status (married/common law, single/never married, divorced/widowed/separated), highest educational attainment (high school or lower, postsecondary), racialized background (yes, no) and household income quintile.

In line with other analyses,^42^-^44^ we coded the age of adults into four groups: young adults (18–34 years), younger middle-aged adults (35–49 years), older middle-aged adults (50–64 years) and older adults (65+ years). We grouped marital status and highest educational attainment into broad categories following previous analyses^45^ and given the size of the sexual minority groups. There were minor discrepancies in how we coded racialized background due to different derived variables provided by Statistics Canada in each dataset at the time of analysis (see the Table 1 notes for more information).

**Table 1:** Sociodemographic characteristics, 2019 CCHS Annual Component and Rapid Response on PMH

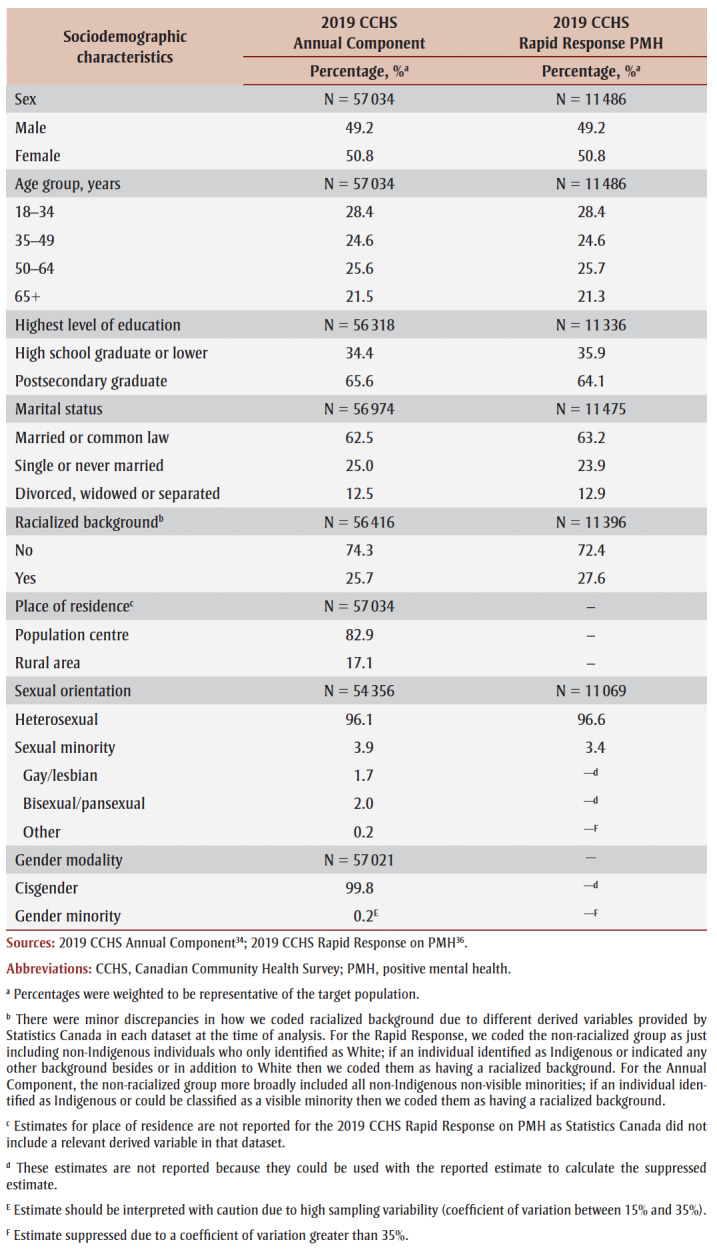

Household income data were obtained by Statistics Canada from linked tax records, imputations or self-reports. Consistent with recommendations from Statistics Canada and given that income can have a nonlinear association with PMH outcomes,^46^ we coded the household income values into quintiles. We also included place of residence (population centre, rural area) as a covariate in the analyses except for the one involving psychological well-being because it was not provided as a derived variable in the Rapid Response dataset by Statistics Canada. Population centres were defined by Statistics Canada as continuously built-up areas with populations of 1000+ and densities of 400+ per km^2^. Due to small sample sizes, we do not report follow-up logistic or linear regression analyses that control for covariates in comparisons involving specific sexual minority groups or gender minority adults.

All estimates were adjusted using sampling weights provided by Statistics Canada and variance was estimated using the bootstrap resampling method with 1000 replications. The sampling weights take into account non-response during the recruitment phase and non-sharing of data with PHAC and Health Canada by respondents. We dealt with missing data by using pairwise deletion to maximize the sample size for each analysis. Estimates with CVs between 15% and 35% (flagged with an ^“^E”) should be interpreted with caution due to high sampling variability; estimates with CVs above 35% (flagged with an ^“F”^) are suppressed. Analyses were conducted in SAS Enterprise Guide version 7.1 (SAS Institute, Cary, NC, USA).

## Results

Based on the Annual Component data, 0.2%^E^ of adults in the Canadian provinces in 2019 were a gender minority and 3.9% were a sexual minority, with 1.7% identifying as gay/lesbian, 2.0% as bisexual/pansexual and 0.2% as another sexual orientation (Table 1).

***Sexual orientation and PMH***

Sexual minority adults reported lower mean life satisfaction (*B* = −0.7, 95% CI: −0.8, −0.5) and were less likely to report high SRMH (OR = 0.4, 95% CI: 0.3, 0.5), high levels of happiness (OR = 0.4, 95% CI: 0.3, 0.5), high community belonging (OR = 0.6, 95% CI: 0.5, 0.7) and high psychological well-being (OR = 0.4, 95% CI: 0.3, 0.6) than heterosexual adults (Table 2). These differences were statistically significant even after controlling for covariates.

**Table 2:** PMH outcomes in sexual minority versus heterosexual adults, 2019 CCHS Annual Component and Rapid Response on PMH

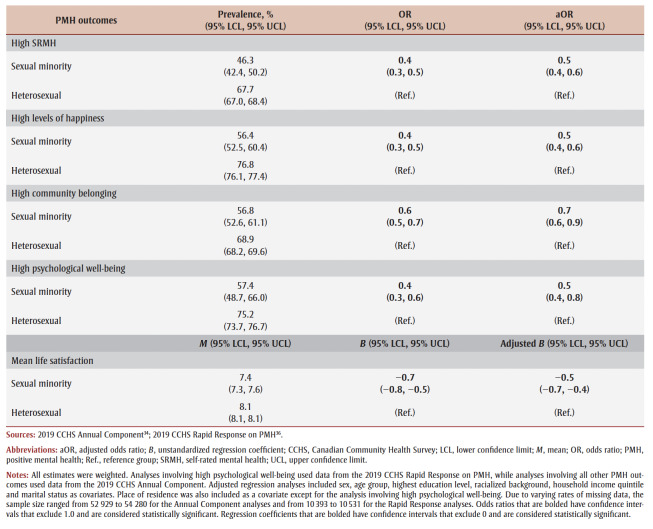

Overall, gay/lesbian and bisexual/pansexual adults reported significantly lower mean life satisfaction (*B* = −0.4, 95% CI: −0.6, −0.2; *B* = −0.9, 95% CI: −1.1, −0.7, respectively) and were significantly less likely to report high SRMH (OR = 0.7, 95% CI: 0.5, 0.9; OR = 0.3, 95% CI: 0.2, 0.4, respectively), high levels of happiness (OR = 0.6, 95% CI: 0.4, 0.8; OR = 0.3, 95% CI: 0.2, 0.4, respectively), high community belonging (OR = 0.6, 95% CI: 0.5, 0.8; OR = 0.6, 95% CI: 0.4, 0.7, respectively) and high psychological well-being (OR = 0.4, 95% CI: 0.2, 0.8^E^; OR = 0.5, 95% CI: 0.3, 0.7, respectively) than heterosexual adults (Table 3).

**Table 3:** PMH outcomes by detailed sexual orientation categories, overall and stratified by sex, 2019 CCHS Annual Component and Rapid Response on PMH

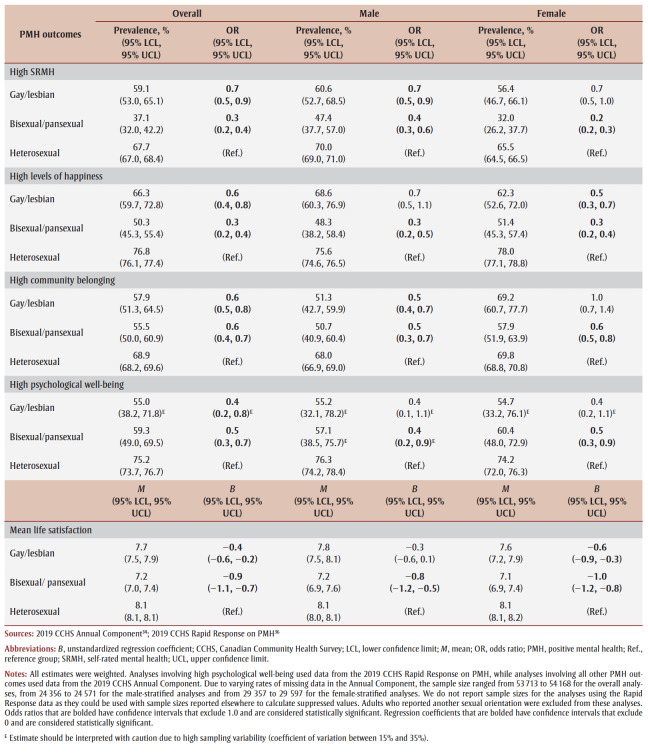

Significant differences across those five PMH outcomes were observed for both bisexual males and bisexual females in the sex-stratified analyses. Gay males were significantly less likely than heterosexual males to report high SRMH (OR = 0.7, 95% CI: 0.5, 0.9) and community belonging (OR = 0.5, 95% CI: 0.4, 0.7), but significant disparities were not observed for high levels of happiness, high psychological well-being or mean life satisfaction. In contrast, compared to heterosexual females, lesbian females reported significantly lower mean life satisfaction (*B* = −0.6, 95% CI: −0.9, −0.3) and were significantly less likely to report high levels of happiness (OR = 0.5, 95% CI: 0.3, 0.7); however, significant disparities were not found for high SRMH, community belonging or psychological well-being (Table 3).

***Gender modality and PMH***

Gender minority adults reported significantly lower mean life satisfaction (*B* = −1.7, 95% CI: −2.6, −0.9) and were significantly less likely to report high SRMH (OR = 0.2, 95% CI: 0.1, 0.5)E and high levels of happiness (OR = 0.2, 95% CI: 0.1, 0.4)^E^ than cisgender adults, but a significant disparity in high community belonging was not observed (Table 4).

**Table 4:** PMH outcomes by gender modality, overall, 2019 CCHS Annual Component

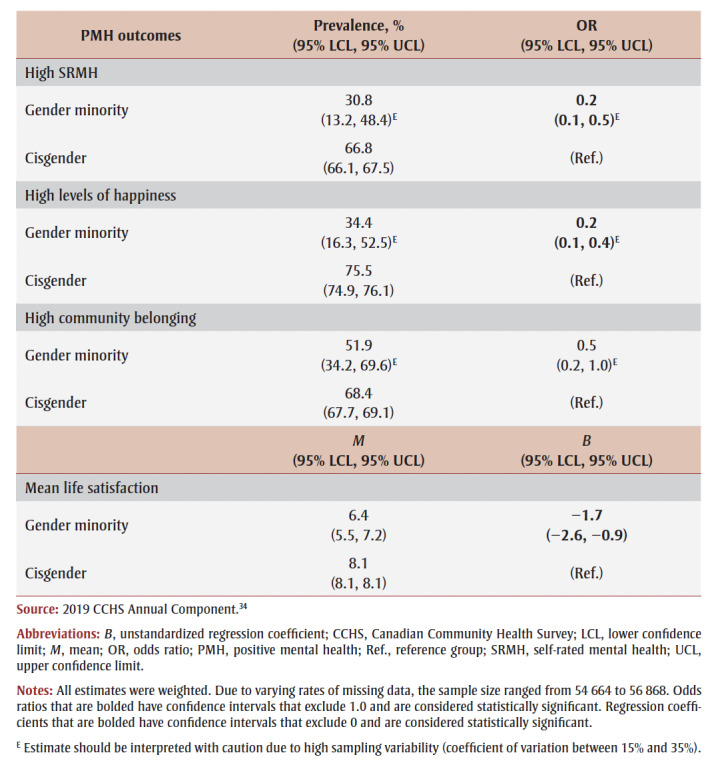

## Discussion

This study documents the PMH of SGM adults across numerous outcomes in Canada in 2019, and investigates disparities in these PMH outcomes compared to heterosexual and cisgender adults. Overall, inequalities in PMH were common. Sexual minority adults reported lower mean life satisfaction and were less likely to report high SRMH, high levels of happiness, high community belonging and high psychological well-being compared to heterosexual adults. Similarly, gender minority adults had lower odds of reporting high SRMH and high levels of happiness, and tended to be less satisfied with life than cisgender adults.

These inequalities tended to be relatively large in magnitude when compared to disparities in PMH outcomes previously observed for other sociodemographic characteristics.^45^ For instance, the percentage difference in high SRMH was 21.4 for sexual minority (vs. heterosexual) individuals and 36.0^E^ for gender minority (vs. cisgender) individuals in the current study, while the percentage difference in high SRMH did not exceed 14.1 in 2019 for comparisons by age group, racialized group membership, immigrant status, household income, place of residence, educational attainment, parental status, living alone, marital status, official language minority or Indigenous identity in previous analyses.^45^ Moreover, the difference in mean life satisfaction was 0.7 for sexual minority (vs. heterosexual) individuals and 1.7 for gender minority (vs. cisgender) individuals in the current study, while the highest mean difference in all the sociodemographic comparisons listed above was 0.6 in 2019.^45^ The especially sizable inequalities in PMH in SGM populations identify a high priority for mental health promotion activities, as well as other interventions aimed at addressing potential determinants of PMH.^47^,^48^

Beyond these overall inequalities, it is also important to acknowledge the heterogeneity that exists within SGM groups. Although PMH tended to be less prevalent among SGM individuals compared to heterosexual and cisgender individuals, there were still large portions of SGM individuals who reported high levels of PMH. For example, high community belonging was reported by the majority of gay, lesbian, bisexual/pansexual and gender minority individuals. Investigations into risk and protective factors that distinguish SGM individuals who report high PMH from those who do not could be important for understanding and promoting individual and community resilience in these populations.^49^ For instance, greater self-compassion appears to be a protective factor as it has been linked to lower minority stress and better well-being among SGM populations.^50^ In contrast, SGM people in Canada are more likely to report experiencing violent victimization,^51^ which is a risk factor of lower PMH;^31^-^33^ safer and more 2SLGBTQ+ friendly communities are likely an important social determinant for these populations and a potential target for more systemic-level interventions.^47^

There were differences in the consistency by which inequalities in PMH outcomes were observed in this study, with disparities between bisexual/pansexual versus heterosexual adults being the most robust. This is in line with previous research findings that the risk of negative psychological outcomes is often highest for bisexual individuals compared to heterosexual or gay/lesbian individuals.^6^,^8^,^10^,^11^,^52^ The distinctive prejudice and discrimination that can be experienced by bisexual people has been offered as an explanation for their heightened risk, including the negative societal attitudes about bisexuality, the invisibility and erasure of bisexual people in wider society, and the lack of affirmative support for bisexual individuals.^52^ Indeed, a recent environmental scan only found one program in Canada that was exclusively dedicated to addressing the social determinants of health among bisexual persons.^47^

Disparities in PMH also tended to be prominent for gender minority adults. Beyond community belonging, only around one-third of gender minority adults reported high SRMH and high levels of happiness, and they rated their life satisfaction 1.7 points lower, on average, than did cisgender individuals. These findings expand previous research on the prevalence of negative psychological outcomes in the transgender population.^7^,^11^ Reducing distal stressors (i.e. being the target of transphobic behaviours) and proximal stressors (i.e. expectations of rejection or discrimination, transgender identity concealment and internalized transphobia) could be important for mental health promotion, as these experiences have been associated with depression and suicidal ideation among gender minority individuals.19 Future research could explore risk and protective factors of PMH for gender minority people.

***Strengths and limitations***

By using data from large population health surveys, we were able to investigate numerous PMH outcomes in the overall SGM populations as well as in specific sexual minority groups. The examination of PMH among gender minority individuals is an especially important contribution as the inclusion of questions asking about both sex and gender is a recent development in Statistics Canada surveys. In addition, our strengths-based focus on PMH allowed us to document that—despite population disparities—many SGM individuals report experiencing well-being in their lives.

Nevertheless, there are limitations that warrant mention. First, we identified many disparities in PMH and offered potential explanations for the results based on minority stress theory and previous research, but we did not directly examine why the disparities exist. The inequalities across the PMH outcomes persisted for sexual minority adults compared to heterosexual adults when we statistically controlled for various sociodemographic characteristics; however, distinct groups were broadly coded into one category for some of the covariates and only unadjusted regression analyses for PMH outcomes were reported for comparisons involving specific sexual minority groups and gender minority adults due to small sample sizes. The small sample sizes also resulted in some relatively wide CIs and likely affected the statistical power to detect significant differences. In addition, the small number of gender minority adults in the dataset restricted our ability to examine specific gender identities (e.g. transgender men, transgender women, nonbinary individuals). The oversampling of SGM individuals in future population health surveys could allow for more comprehensive examinations of specific SGM identities, as well as the disaggregation of results by other potentially important sociodemographic factors.^53^ For instance, age breakdowns could be informative; experiences of discrimination and disparities in mental health have been found to vary across the life course among sexual minority individuals in other countries.^54^,^55^

Self-reported responses to questionnaires may be subject to recall bias and social desirability bias.^56^ Further, the unwillingness of some respondents to disclose their sexual orientation or gender modality could have resulted in some misclassification.^57^ While respondents were asked to report on their sexual identity, there are other important dimensions of sexual orientation that could have been assessed (i.e. sexual attraction and sexual behaviour).^53^,^58^ Moreover, the survey question on gender included “male” and “female” as response options instead of the more relevant “man” and “woman.”^59^ Finally, we may be missing data from the most at-risk SGM individuals (e.g. those who are experiencing homelessness).^60^

## Conclusion

We found that PMH tended to be less common among SGM adults than among heterosexual and cisgender adults in 2019. Future research could explore the mechanisms by which SGM people experience lower PMH, risk and protective factors of PMH in SGM populations, how PMH might depend on the interaction between sexual orientation and gender modality with other sociodemographic characteristics, and how the observed disparities in PMH may have changed over time.

## Acknowledgements

We would like to thank Raelyne Dopko and Elia Palladino for their feedback in the early stages of planning this project, and Mlanie Varin for her feedback at multiple stages of the project and for re-running analyses to double-check results.

## Conflicts of interest

The authors have no conflicts of interest.

## Authors’ contributions and statement

SH: Writing – original draft, writing – review & editing.

CAC: Conceptualization, methodology, writing – original draft, writing – review & editing.

LL: Methodology, formal analysis, writing – review & editing.

All authors approved the manuscript for publication.

The content and views expressed in this article are those of the authors and do not necessarily reflect those of the Government of Canada.
